# Efficient light transfer in coupled nonlinear triple waveguides using shortcuts to adiabaticity

**DOI:** 10.1038/s41598-023-28427-0

**Published:** 2023-01-24

**Authors:** Vasileios Evangelakos, Emmanuel Paspalakis, Dionisis Stefanatos

**Affiliations:** Materials Science Department, School of Natural Sciences, University of Patras, Patras, 26504 Greece

**Keywords:** Optical physics, Optics and photonics, Quantum optics

## Abstract

We use the method of shortcuts to adiabaticity to design the variable couplings in a three-waveguide directional coupler which may contain nonlinear elements, in order to accomplish efficient light transfer between the outer waveguides for shorter device lengths, despite the presence of nonlinearity. The shortcut couplings are obtained for the ideal case where all the waveguides are linear, for which a perfect transfer is guaranteed in theory, but are tested for various combinations of linear and nonlinear waveguides in the device. We show with numerical simulations that, in most configurations, high levels of transfer efficiency can be maintained even for large values of the input power, and for shorter lengths than those of conventional adiabatic devices. We also find that efficiency is improved for shortcut couplings with less spatial extent, since in this case the nonlinearity acts during a shorter range. The present work is expected to find application in research fields like optoelectronic computing and ultrafast light switching, where the fast and controlled light transmission inside a set of waveguides is a crucial task. Additionally, the reduction in the device size may be exploited for incorporating them in integrated optical systems, where a high density of waveguides is required.

## Introduction

Studying the properties of waveguide directional couplers constitutes a very active research area within the fields of photonics and optoelectronics^1^. The reason behind this intense interest is that such devices may be exploited as beam splitters, switches, Mach-Zehnder interferometers, optical ring resonators, and other parts of components appearing in a variety of optical integrated circuits. The most simple directional coupler arrangement consists of two parallel coupled waveguides at a short distance between them. In this device, light travels in a periodic manner from the one waveguide to the other, as it propagates along them.

In order to improve the robustness properties of the above simple directional coupler, more sophisticated configurations have been suggested through the years. Many of these designs rely on the use of varying coupling and propagation coefficients^2–6^. In these configuration the switching of light between the waveguides relies on the adiabatic (slow) evolution of a normal mode (eigenstate) of the system. The advantage of such devices lies on the fact that adiabatic evolution is robust against small to modest deviations in the system parameters (e.g. errors during the layout implementation). A three-waveguide directional coupler relying on the adiabatic evolution of system’s “dark” state was put forward^4,5,7^, essentially suggesting the optical analogue of stimulated Raman adiabatic passage (STIRAP), an extremely successful method for high fidelity population transfer in quantum systems^8,9^. If the varied coupling coefficients are applied in the counter-intuitive order and also satisfy the adiabaticity condition, then light is efficiently transmitted between the first and third outermost waveguides, while the intermediate waveguide is hardly excited. The aforementioned adiabatic directional coupler was initially investigated experimentally by Longhi et al.^10^. The basic idea for adiabatic design of the varying coupling coefficients has been exploited in many other coupler layouts, in both theoretical^11–14^ and experimental^15,15–18^ works. An analogous technique has been suggested in periodic (grating-assisted) directional waveguide couplers^19–21^, in a device composed of three coupled waveguides with curved axes^22^, as well as for adiabatic mode switching in multimode waveguides by making use of computer-generated planar holograms^23^. Finally, different works^24,25^, presented methods inspired by adiabatic elimination in quantum physics was proposed for the transfer of light between the outer waveguides in a waveguide array.

Despite the advantages of optical devices which exploit adiabatic propagation, including the broadband range of supported frequencies and also the tolerance against moderate variations in device parameters, there is also an important drawback. In order to satisfy the conditions which ensure the adiabatic propagation of light, the length of the corresponding devices should be sufficiently large. This puts a major obstacle in using such devices in integrated optical systems, where a high density of waveguides is required. For this reason many efforts have been made to improve the geometry and reduce their size^3,26–32^, however, the lack of a simple set of design rules made it difficult to choose the appropriate modification for each application. An additional problem arises for the important class of directional waveguide couplers with waveguides containing nonlinear optical materials^33–46^. Note that the particular research interest for these devices follows from their all-optical switching applications^33^. Lahini et al.^16^ thoroughly investigated the influence of optical nonlinearity on the light transfer properties in an adiabatic three-waveguide directional coupler. They demonstrated both in theory and experiment that for materials whose refractive index depends on the intensity of light (optical Kerr effect), increasing the power of light inserted into the device results in a decrease in the transfer efficiency between the outer waveguides, because of the destruction of the “dark” eigenstate.

In order to accelerate adiabatic propagation and thus reduce the size of the corresponding directional couplers, a collection of methods referred as *shortcuts to adiabaticity*^47^ have been employed. Originally developed in the context of quantum control theory, these methods were quickly adopted for the efficient design of optical devices by exploiting the analogy between quantum mechanics and waveguide optics^48^, according to which the propagation of light along arrays of coupled waveguides is equivalent to the time evolution of a multilevel quantum system. The main idea behind these methods is to reach the same final state as the slow adiabatic evolution, without necessarily tracking the instantaneous adiabatic eigenstate at each moment. In order to accomplish a shortcut to the adiabatic propagation of light following the transitionless tracking algorithm^49–53^, in Refs.^54,55^ a driving opposite to the non-adiabatic evolution of the system was added to the couplings between the waveguides. In the above works, however, the counterdiabatic terms in the couplings turned out to be complex and thus inapplicable. The problem was resolved in Ref.^56^ using Lie transformation theory, in order to modify the terms which cancel the non-adiabatic evolution and make them real and physically applicable. The same technique was later used to realize beam splitters consisting of a three-waveguide array^57^. Another shortcut method used to accelerate adiabatic propagation is reverse engineering^58^ based on the Lewis-Riesenfeld invariant theory^59^. It was used to improve light transferring in systems consisting of multi-mode waveguides^60,61^ or arrays of several coupled waveguides^62^. It also led to the realization of three-waveguide directional couplers^63^ and quantum logic gates based on waveguides^64^. The two shortcut techniques discussed above, although seem to be quite different, are actually equivalent approaches with different parameterization^65^. Based on these previous works, several protocols have emerged that are used to accelerate adiabatic propagation and studies are being carried out that apply these protocols to devices that function as beam splitters^66,67^ or directional couplers^68,69^. Recent reviews on the applications of shortcut to adiabaticity methods in optical waveguides can be found in Refs.^70,71^.

In this work we use the transitionless tracking algorithm to design the varying couplings in a three-waveguide directional coupler which may contain nonlinear elements, in order to achieve efficient light transfer between the outer waveguides for shorter device lengths and despite the presence of nonlinearity. The shortcut couplings are derived for the ideal case where all the waveguides are linear, for which a perfect transfer is assured, but are tested for various combinations of linear and nonlinear waveguides in the device. We show with numerical simulations that, in most configurations, high levels of transfer efficiency can be maintained even for large values of the input power, and for shorter lengths than those needed by conventional adiabatic devices. We also find that the efficiency is improved for shortcut couplings with less spatial extent, since in this case the nonlinearity acts during a shorter interval. The best performance is obtained for the symmetric configurations where the nonlinearity is present only in the middle or only in the two outer waveguides. The reason is that in the first case the dark state is still an eigenstate of the system, while in the second case the effective two-photon detuning is reduced. The worst performance for increasing input power is observed for the nonsymmetric configurations where the nonlinearity is present in the first (input) waveguide, thus it is immediately activated and undermines the transfer.

## Nonlinear triple waveguide

**Figure 1:**
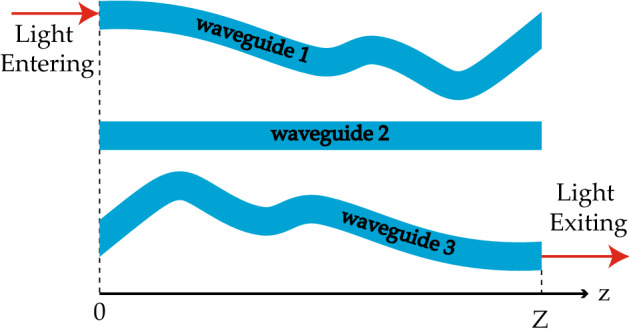
Schematic illustration of the nonlinear triple waveguide.

A schematic illustration of the directional coupler under study is given in Fig. 1. The propagation of light in a system of three coupled nonlinear waveguides with the same transmission coefficients $\beta$ is described by the following equation^16^

1 $$\begin{aligned}  -i {\frac{d}{dz}}b(z) = M(z)b(z),\end{aligned} $$

where

2 $$\begin{aligned} b(z)= \left( \begin{array}{c} b_1(z) \\ b_2(z) \\ b_3(z) \end{array} \right) \end{aligned}$$

is the vector with components the field amplitudes in the waveguides, expressed as functions of the propagation distance *z*, and *M*(*z*) is the transmission matrix given by the expression

3 $$\begin{aligned} M(z)= \left( \begin{array}{ccc} Q_1|b_1(z)|^2 &{} k_{12}(z) &{} 0 \\ k_{12}(z) &{} Q_2|b_2(z)|^2 &{} k_{23}(z) \\ 0 &{} k_{23}(z) &{} Q_3|b_3(z)|^2 \end{array} \right) . \end{aligned}$$

Note that here we have omitted the common transmission coefficient, since it simply adds a common phase factor to the field amplitudes. On the other hand, $k_{12}$ and $k_{23}$ are the coupling coefficients between the waveguides 1-2 and 2-3, respectively, while $Q_i=(2\pi n_i^{(2)})/(\lambda A_i^{(eff)})$^72^ are the nonlinearity coefficients which are proportional to the nonlinear refractive index $n_i^{(2)}$ and inversely proportional to the effective cross section $A_i^{(eff)}$ of each waveguide. Typical values of these parameters are given in the first paragraph of the section where we present the simulation results. Note that the total refractive index $n_i=n_i^{(0)}+n_i^{(2)}I$ depends on the field intensity *I*, and this is the physical origin of the nonlinearity in system (3). By carefully selecting the functions $k_{12}(z), k_{23}(z)$ we can manipulate the light distribution between the waveguides, as it propagates along them. The variation of the coupling coefficients with the propagation distance can be achieved using properly designed gratings^54^ or by appropriately varying the distance between the corresponding waveguides^65^ as shown in Fig. 1, see also the recent reviews^70,71^.

## Light transfer in the absence of nonlinearity

In this section we describe how the coupling coefficients can be designed for the efficient transfer of light entering the first waveguide to the third waveguide. The coupling coefficients will be obtained for the ideal case where $Q_1=Q_2=Q_3=0$, but their performance will be tested in the presence of nonlinearity with nonzero $Q_i$ in the next section. We start by quickly reminding the reader the adiabatic transfer method and then present the shortcut to adiabaticity method.

### Adiabatic transfer

In the absence of nonlinearity ($Q_i=0$), but also describing the case where the input power is small, the transmission matrix (3) becomes

4 $$\begin{aligned} M_0(z)= \left( \begin{array}{ccc} 0 &{} k_{12}(z) &{} 0 \\ k_{12}(z) &{} 0 &{} k_{23}(z) \\ 0 &{} k_{23}(z) &{} 0 \end{array} \right) \end{aligned}$$

In this case, the propagation equation (1) is analogous to the Schrödinger equation for a three-level $\Lambda$-type quantum system. The light transfer from the first to the third waveguide is analogous to the population transfer from the first to the third level in this quantum system, thus Stimulated Raman Adiabatic Passage (STIRAP) can be used for the derivation of the appropriate coupling coefficients^5,8,9^. We briefly explain how it can be done. The three-waveguide problem with transmission matrix (4) can be reduced to a two-waveguide problem with matrix

5 $$\begin{aligned} M'_0(z)= \frac{1}{2}\left( \begin{array}{cc} k_{23}(z) &{} k_{12}(z) \\ k_{12}(z) &{} -k_{23}(z) \end{array} \right) =\frac{1}{2}(k_{12}\sigma _x+k_{23}\sigma _z), \end{aligned}$$

where the original field amplitudes ${\varvec{b}}(z)=[b_1(z) \, b_2(z) \, b_3(z)]^T$ are connected to those of the simplified system ${\varvec{c}}(z)=[c_1(z) \, c_2(z)]^T$ through the transformation

6 $$\begin{aligned} \begin{array}{ccc} b_1(z)&{}=&{}|c_1(z)|^2-|c_2(z)|^2 \\ b_2(z)&{}=&{}2i\text{ Im } \left[ c_1^*(z)c_2(z)\right] \\ b_3(z)&{}=&{}-2\text{ Re } \left[ c_1^*(z)c_2(z)\right] \end{array}. \end{aligned}$$

If the coupling coefficients are parameterized as follows

7 $$\begin{aligned} \begin{array}{c} k_{12}=k(z)\sin {\theta (z)} \\ k_{23}=k(z)\cos {\theta (z)} \end{array}, \end{aligned}$$

where $k(z)=\sqrt{k_{12}^2(z)+k_{23}^2(z)}$ and $\tan {\theta (z)}=k_{12}(z)/k_{23}(z)$, then the two-waveguide system has the following eigenstates

8a $$\begin{aligned} |\phi _+(z)\rangle= & {} \left( \begin{array}{c} \cos {\frac{\theta (z)}{2}}\\ \sin {\frac{\theta (z)}{2}} \end{array}\right) , \end{aligned}$$

8b $$\begin{aligned} |\phi _-(z)\rangle= & {} \left( \begin{array}{c} \sin {\frac{\theta (z)}{2}}\\ -\cos {\frac{\theta (z)}{2}} \end{array}\right) , \end{aligned}$$

with corresponding eigenvalues $E_{\pm }(z)=\pm k(z)/2$. By slowly changing the mixing angle $\theta (z)$ of the coupling coefficients from $\theta (0)=0$, the two-waveguide system evolves along the eigenstate $|\phi _+(z)\rangle$, and thus amplitudes $c_1(z), c_2(z)$ follow Eq. (8a). The initial condition on the angle ensures that $|\phi _+(0)\rangle =(1 \; 0)^T$, corresponding to light entering from the first waveguide. From transformation (6) we easily find that in this case the original three-waveguide system evolves along the “dark” state

9 $$\begin{aligned} |\psi _d(z)\rangle =\cos {\theta (z)}|1\rangle -\sin {\theta (z)}|3\rangle , \end{aligned}$$

where only the first and third waveguides have nonzero field amplitudes, since any fraction of light entering the second waveguide is immediately transferred to the third one. From this expression it is obvious that, if we choose the function $\theta (z)$ to satisfy the terminal condition $\theta (Z)=\pi /2$ at the final distance $z=Z$, then light travels from the first waveguide initially to the third waveguide at the end of the propagation distance *Z*. The necessary condition for this light transfer to be adiabatic is $\sqrt{k_{12}^2(z)+k_{23}^2(z)}\Delta z\gg 1$, where $\Delta z$ is the distance for which both $k_{12}(z), k_{23}(z)$ are nonzero, thus strong coupling and enough space overlapping between the coefficients is required^5,8,9^.

**Figure 2:**
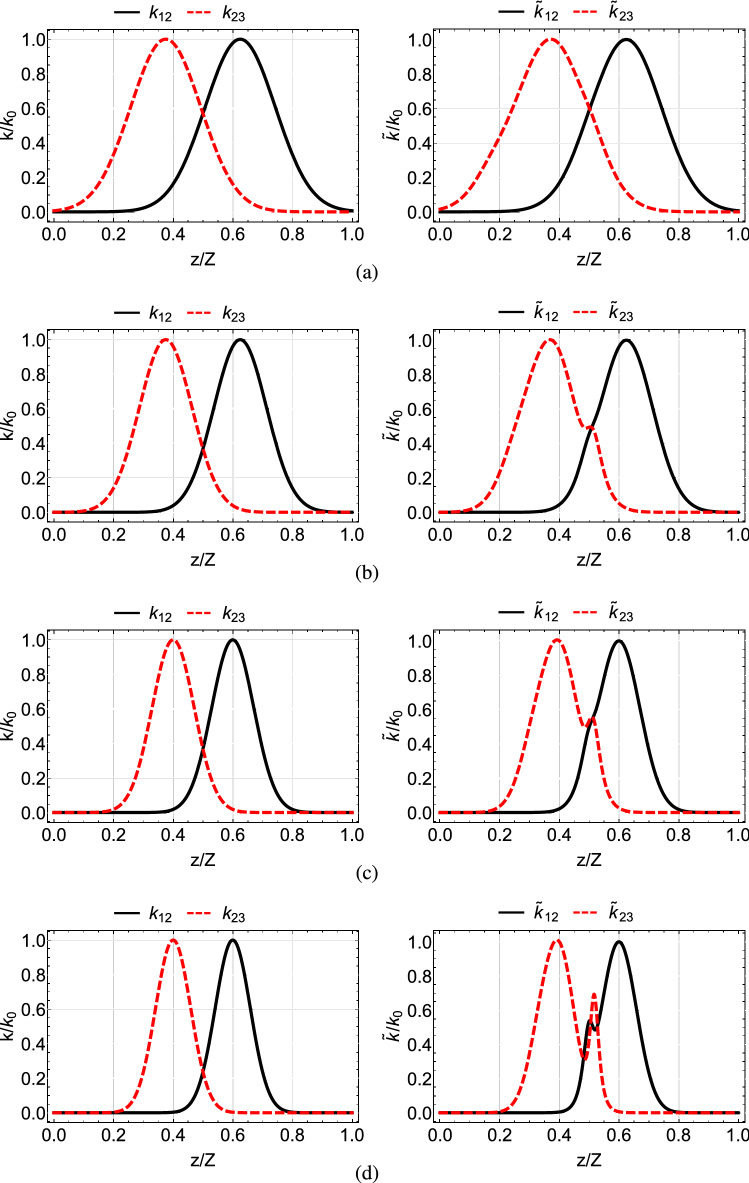
Gaussian (left column) and modified shortcut (right column) coupling coefficients for parameters $Z=30$ mm, $k_0=2$ mm$^{-1}$ and (**a**) $\tau =Z/8$ and $\zeta =Z/6$, (**b**) $\tau =Z/8$ and $\zeta =Z/8$, (**c**) $\tau =Z/10$ and $\zeta =Z/10$, (**d**) $\tau =Z/10$ and $\zeta =Z/12$.

**Figure 3:**
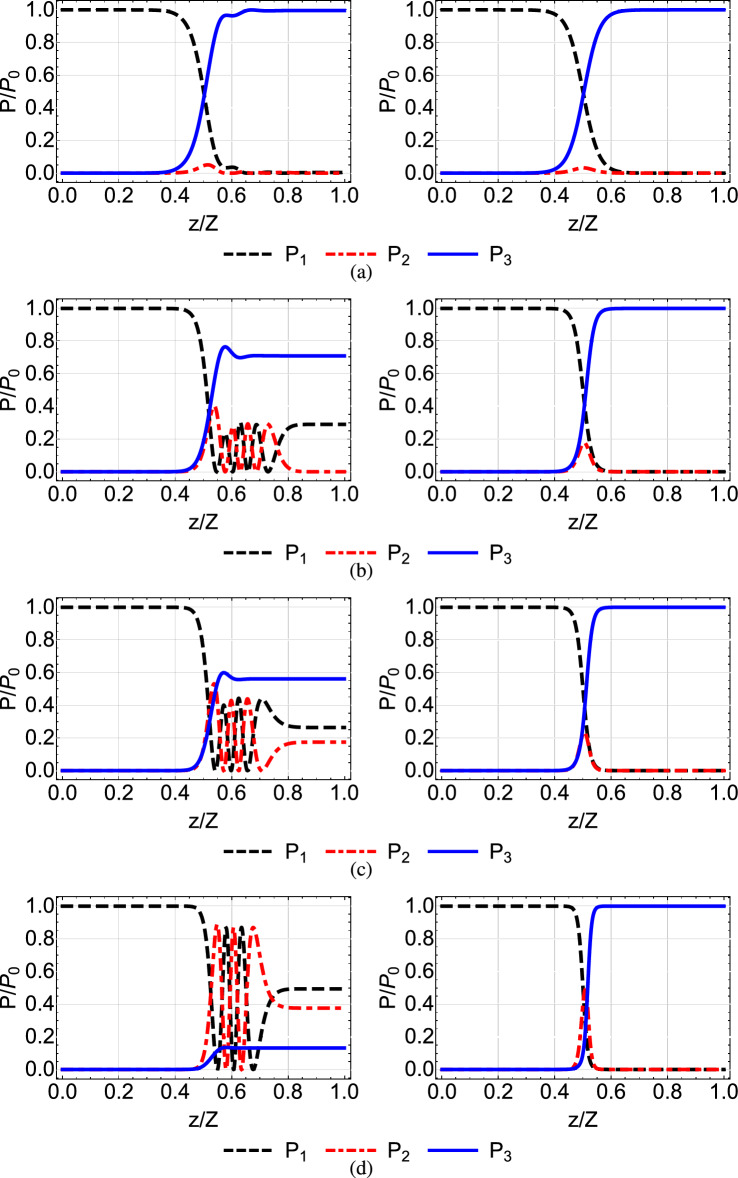
Normalized power $P_i(z)/P_0$ at each waveguide as a function of normalized distance, for the Gaussian (left column) and modified shortcut (right column) coupling coefficients of Fig. 2.

A popular choice is the coupling coefficients to have the following Gaussian form

10 $$\begin{aligned} \begin{array}{ccc} k_{12}&{}=&{}k_{0}e^{-\left( \frac{z-Z/2-\tau }{\zeta }\right) ^2}, \\ k_{23}&{}=&{}k_{0}e^{-\left( \frac{z-Z/2+\tau }{\zeta }\right) ^2}, \end{array} \end{aligned}$$

where $k_{0}$ is the maximum value of the coupling coefficients, $\zeta$ denotes the width of the couplings and $2\tau$ is the spatial separation between their peaks. By selecting those parameters appropriately, we can approximately satisfy the above boundary conditions. For $\tau >0$, corresponding to the counterintuitive pulse order of STIRAP where the $k_{23}$ coupling is activated before the $k_{12}$ coupling, and with properly choosing parameters $k_{0}, \tau , \zeta$, the boundary conditions $\theta (0)=0, \theta (Z)=\pi /2$, as well as the adiabaticity condition can be satisfied. In the left column of Fig. 2 we display Gaussian coupling coefficients for $Z=30$ mm, $k_0=2$ mm$^{-1}$ and various values of parameters $\tau , \zeta$, while in the left column of Fig. 3 we plot the normalized power $P_i(z)/P_0$, $i=1, 2, 3$ in each waveguide, as a function of normalized distance *z*/*Z*. We observe that for the combination of coupling coefficients shown in the left panel of the top row in Fig. 2, the light transfer to the third waveguide is accomplished with high fidelity, thus the adiabaticity condition is satisfied. As we move from the top to the bottom of the left column in Fig. 2, we observe that the coupling coefficients are more narrow and there is less overlap between them. This results in the violation of the adiabaticity condition and the incomplete light transfer to the third waveguide, as displayed in the lower three panels of the left column of Fig. 3. The adiabaticity condition for these cases can be restored by increasing the value of $k_{0}$, i.e. using larger coupling coefficients, or by increasing the absolute propagation length *Z*. In the following subsection we explain how light transfer to the third waveguide can be effectively accomplished by modifying the shape of the coupling coefficients, without increasing $k_{0}$ or *Z*.

### Shortcut to adiabaticity

When the condition for adiabatic transfer is not met, in order to retain the desired light transition we must introduce a counterdiabatic term in our transmission matrix which, for the equivalent two-waveguide system has the form^49–53^

11 $$\begin{aligned} M'_{cd}(z)=&i\sum _{n=\pm } \bigg [|{\dot{\phi }}_n(z)\rangle \langle \phi _n(z)|\nonumber \\&-\langle \phi _n(z)|\dot{\phi }_n(z)\rangle |\phi _n(z)\rangle \langle \phi _n(z)|\bigg ]. \end{aligned}$$

Since $|{\dot{\phi }}_{\pm }(z)\rangle =\mp |\phi _{\mp }(z)\rangle$, we eventually get

12 $$\begin{aligned} M'_{cd}(z)=\left( \begin{array}{cc} 0 &{} -i\frac{{\dot{\theta }}(z)}{2}\\ i\frac{{\dot{\theta }}(z)}{2} &{} 0 \end{array}\right) =\frac{{\dot{\theta }}(z)}{2}\sigma _y, \end{aligned}$$

where the dot denotes the derivative with respect to the propagation distance *z*. This counterdiabatic term, proportional to the Pauli matrix $\sigma _y$, introduces in the original three-waveguide system a direct coupling between the first and third waveguides. In order to retain the original configuration, where only the waveguides 1-2 and 2-3 are coupled, the following unitary transformation can be applied on the state (field amplitudes) of the two-waveguide system^52,53^

13 $$\begin{aligned} U(z)=e^{-i\frac{\phi (z)}{2}\sigma _z}=\left( \begin{array}{cc} e^{-i\frac{\phi (z)}{2}} &{} 0\\ 0 &{} e^{+i\frac{\phi (z)}{2}} \end{array}\right) . \end{aligned}$$

The modified transmission matrix for the equivalent two-waveguide system, after the addition of the counterdiabatic term and the application of the unitary transformation, becomes

14 $$\begin{aligned} {\tilde{M}}'= & {} U^{\dag }(M_0'+M'_{cd})U+i{\dot{U}}^{\dag }U\nonumber \\= & {} \frac{1}{2}(k_{12}\cos {\phi }+\dot{\theta }\sin {\phi })\sigma _x\nonumber \\+ & {} \frac{1}{2}(k_{12}\sin {\phi }-\dot{\theta }\cos {\phi })\sigma _y+\frac{1}{2}(k_{23}-\dot{\phi })\sigma _z \end{aligned}$$

In order to eliminate the term proportional to $\sigma _y$, we demand $\phi (z)=\tan ^{-1}{( \dot{\theta }/k_{12})}$. Then, by defining the modified coupling coefficents as

15a $$\begin{aligned} {\tilde{k}}_{12}= & {} k_{12}\cos {(\phi )}+\dot{\theta }\sin {\phi }, \end{aligned}$$

15b $$\begin{aligned} {\tilde{k}}_{23}= & {} k_{23}-\dot{\phi }, \end{aligned}$$

the modified transmission matrix for the equivalent two-waveguide system attains the original form (5)

16 $$\begin{aligned} {\tilde{M}}'=\frac{1}{2}\left( {\tilde{k}}_{12}\sigma _x+{\tilde{k}}_{23}\sigma _z \right) , \end{aligned}$$

with the original couplings replaced by the modified ones. The corresponding modified transmission matrix for the three-waveguide system is

17 $$\begin{aligned} {\tilde{M}}= & {} \left( \begin{array}{ccc} 0 &{} {\tilde{k}}_{12}(z) &{} 0 \\ {\tilde{k}}_{12}(z) &{} 0 &{} {\tilde{k}}_{23}(z) \\ 0 &{} {\tilde{k}}_{23}(z) &{} 0 \end{array} \right) , \end{aligned}$$

which is also similar to the original matrix (4) but again with the modified couplings. The field amplitudes $\tilde{{\varvec{b}}}=({\tilde{b}}_1 \, {\tilde{b}}_2 \, {\tilde{b}}_3)^T$ in the three-waveguide system under transmission matrix (17) are

18 $$\begin{aligned} \begin{array}{ccccc} {\tilde{b}}_1(z)&{}=&{}|{\tilde{c}}_1(z)|^2-|{\tilde{c}}_2(z)|^2&{}=&{}\sqrt{P_0}\cos {\theta (z)},\\ {\tilde{b}}_2(z)&{}=&{}2i\text{ Im }[{\tilde{c}}_1^*(z){\tilde{c}}_2(z)]&{}=&{}i \sqrt{P_0}\sin {\theta (z)}\cos {\phi (z)}, \\ {\tilde{b}}_3(z)&{}=&{}-2\text{ Re }[{\tilde{c}}_1^*(z){\tilde{c}}_2(z)]&{}=&{}-\sqrt{P_0}\sin {\theta (z)}\cos {\phi (z)}, \end{array} \end{aligned}$$

where the field amplitudes in the equivalent two-waveguide system under transmission matrix (16) are obtained from the relation $\tilde{{\varvec{c}}}=({\tilde{c}}_1 \, {\tilde{c}}_2)^T\sim U^{\dag }|\phi _+\rangle$, using Eqs. (13) and (8a), and $P_0$ is the input power.

We next explain how to design^53^ the function $\theta (z)$ in order to accomplish light transfer from the first to the third waveguide under the modified transmission matrix (17) and thus the evolution given in Eq. (18). Note that by definition angle $\phi$ is expressed in terms of $\theta , \dot{\theta }$, thus any requirement on this may also be translated to a condition on $\theta$. The normalized power inside each waveguide is $P_i/P_0=|{\tilde{b}}_i(z)|^2/P_0$, $i=1, 2, 3$. All the light is inserted at $z=0$ to the first waveguide, thus $|{\tilde{b}}_1(0)|^2=P_0$, which is translated into the condition $\theta (0)=0$. At the end of the propagation length, $z=Z$, the light should be completely transferred to the third waveguide, which is accomplished if we take $\theta (Z)=\frac{\pi }{2}$ and $\phi (Z)=0$. From the definition of angle $\phi$ we see that the latter condition is in general satisfied by setting $\dot{\theta }(Z)=0$. We also require ${\tilde{k}}_{12}(0)=0$ and ${\tilde{k}}_{23}(Z)=0$, so the initial and final states are stationary states of the equation $-i\dot{\tilde{{\varvec{b}}}}={\tilde{M}}\tilde{{\varvec{b}}}$. Using Eqs. (15), (7), and the definition of $\phi$, it is not hard to verify that these conditions can be satisfied if we additionally require $\dot{\theta }(0)=\ddot{\theta }(Z)=0$.

If we consider original coupling coefficients $k_{12}, k_{23}$ with the Gaussian shape (10), then from Eq. (7) we get

19 $$\begin{aligned} \tan {\theta }=e^{\frac{4\tau }{\zeta ^2}(z-Z/2)}. \end{aligned}$$

For this form of pulses the boundary conditions for $\theta$ are not exact, but can be fulfilled to an excellent approximation by the appropriate choice of the pulse parameters^53^. In the right column of Fig. 2 we plot the modified couplings for shortcut light transfer obtained from the Gaussian original couplings of the left column. In the right column of Fig. 3 we display the corresponding normalized power in each waveguide. We observe that with the modified couplings a perfect light transfer to the third waveguide is accomplished, even for the cases where the original couplings are more narrow and fail, see the three lower rows of Fig. 3. The spatial range over which the modified couplings are nonzero, which essentially determines the device length, covers a smaller portion of the propagation distance *Z*, compare for example the right panel of Fig. 2d with the left panel of Fig. 2a, thus lowering the requirement on the coupler length. The success of the modified couplings can be understood by carefully inspecting Fig. 2. There, in the left column, we notice that the overlapping region between the original Gaussian coefficients is reduced as we move from top to bottom. In the right column displaying the modified couplings we observe that the overlapping region is also reduced as we move from top to bottom, but at the same time the couplings in this region are increased, resulting in the successful transfer of light despite the limited spatial overlap. Another useful observation which can be made from the right column of Fig. 3 is that, as we move from top to bottom and the spatial extension of the coupling coefficients is reduced, the light transfer from the first to the third waveguide is accomplished with more power passing through the second (intermediate) waveguide (red dashed-dotted line).

## Numerical results in the presence of nonlinearity

**Figure 4:**
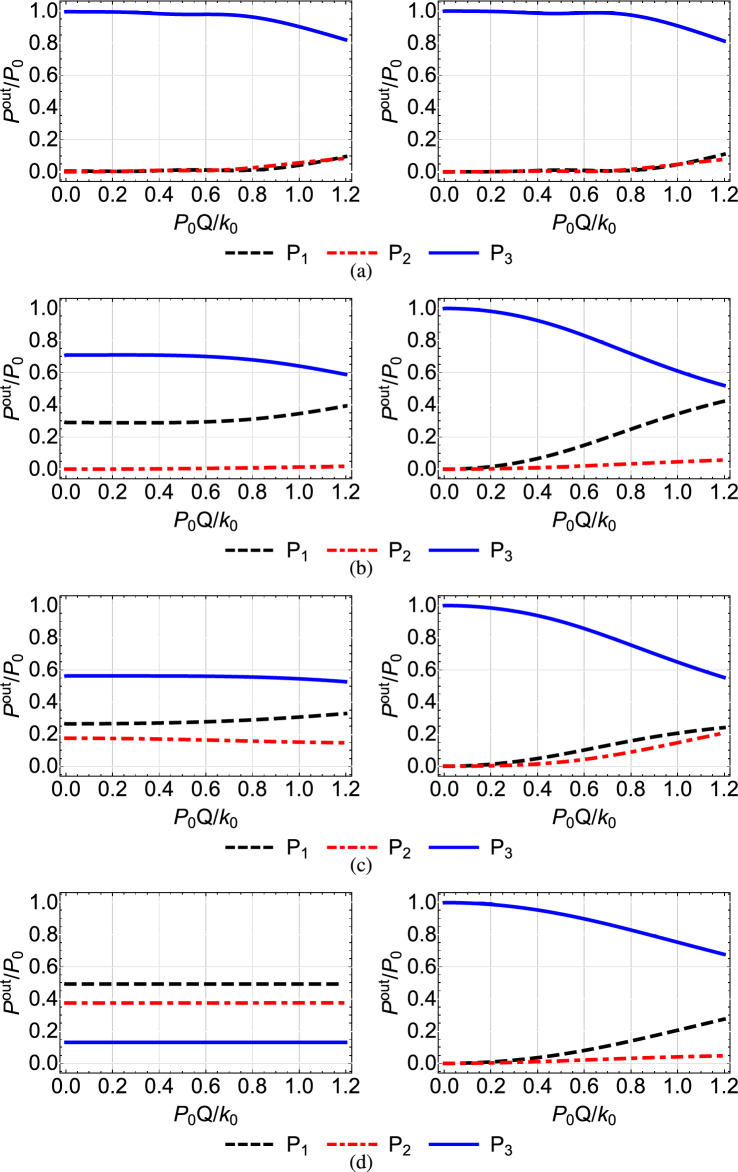
Normalized output power $P_i^{out}/P_0=P_i(Z)/P_0$ at each waveguide as a function of the normalized input power $P_0Q/k_0$ when $Q_1=0, Q_2=0, Q_3=Q$, for the Gaussian (left column) and modified shortcut (right column) couplings displayed in the left and right columns of Fig. 2, respectively.

**Figure 5:**
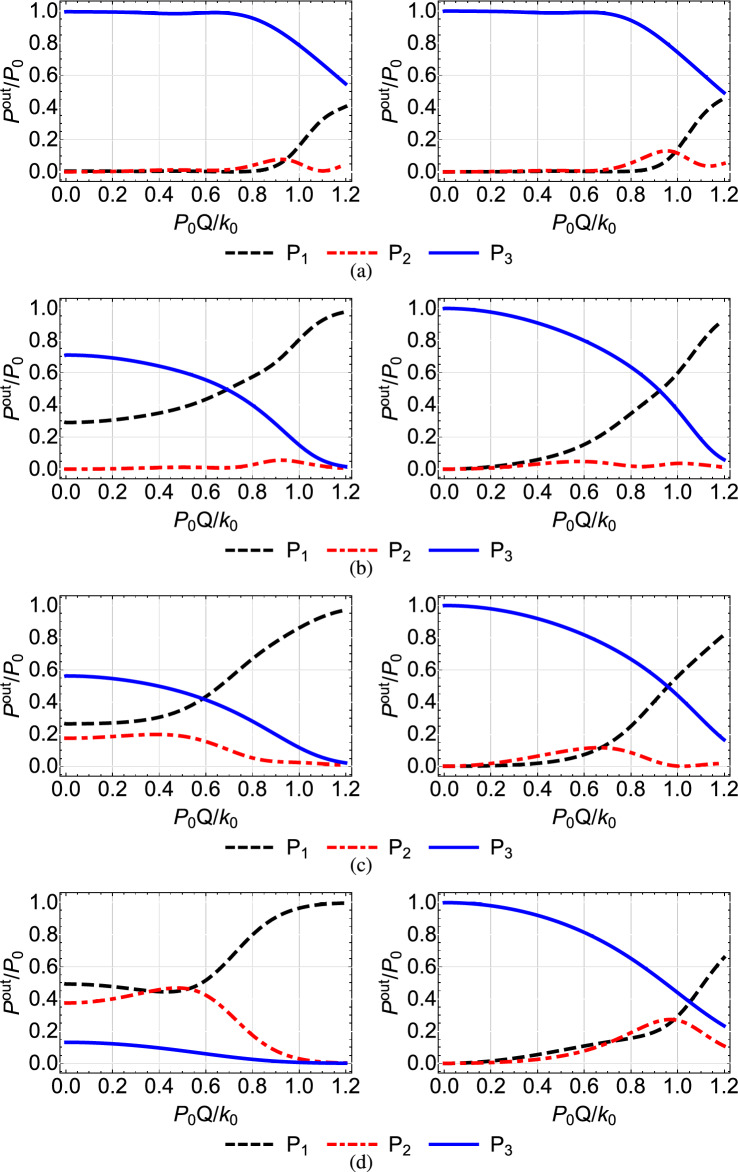
Normalized output power $P_i^{out}/P_0=P_i(Z)/P_0$ at each waveguide as a function of the normalized input power $P_0Q/k_0$ when $Q_1=Q, Q_2=0, Q_3=0$, for the Gaussian (left column) and modified shortcut (right column) couplings displayed in the left and right columns of Fig. 2, respectively.

**Figure 6:**
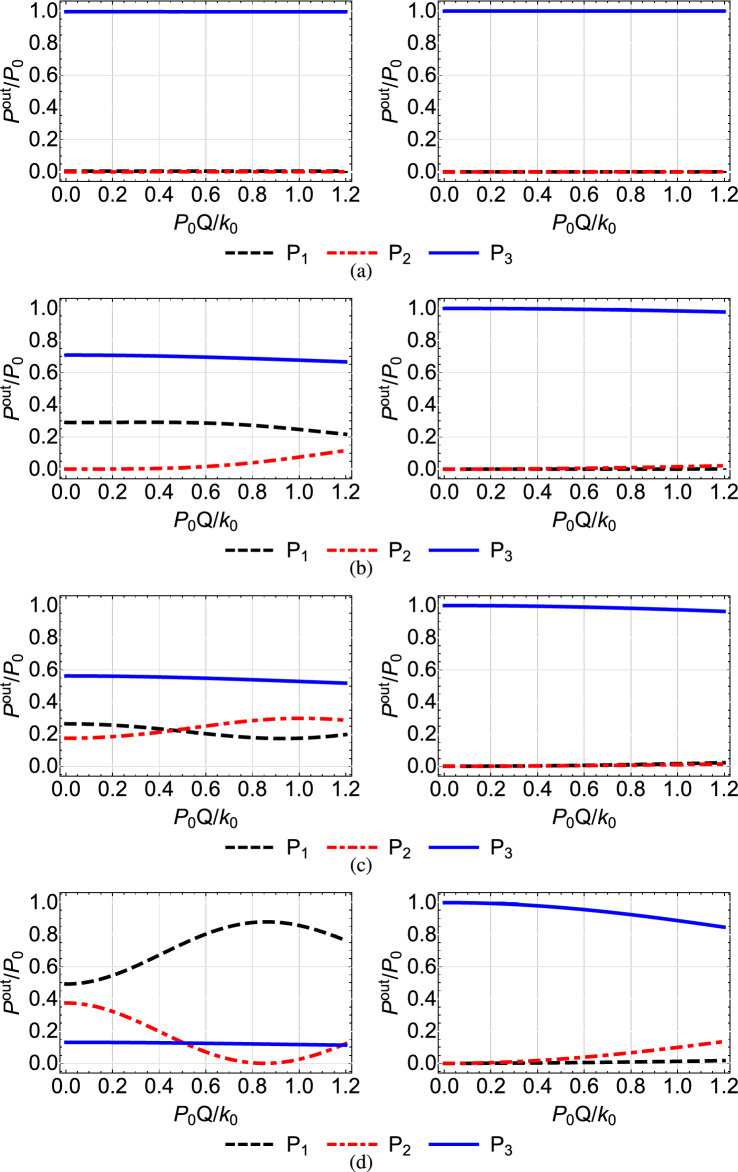
Normalized output power $P_i^{out}/P_0=P_i(Z)/P_0$ at each waveguide as a function of the normalized input power $P_0Q/k_0$ when $Q_1=0, Q_2=Q, Q_3=0$, for the Gaussian (left column) and modified shortcut (right column) couplings displayed in the left and right columns of Fig. 2, respectively.

**Figure 7:**
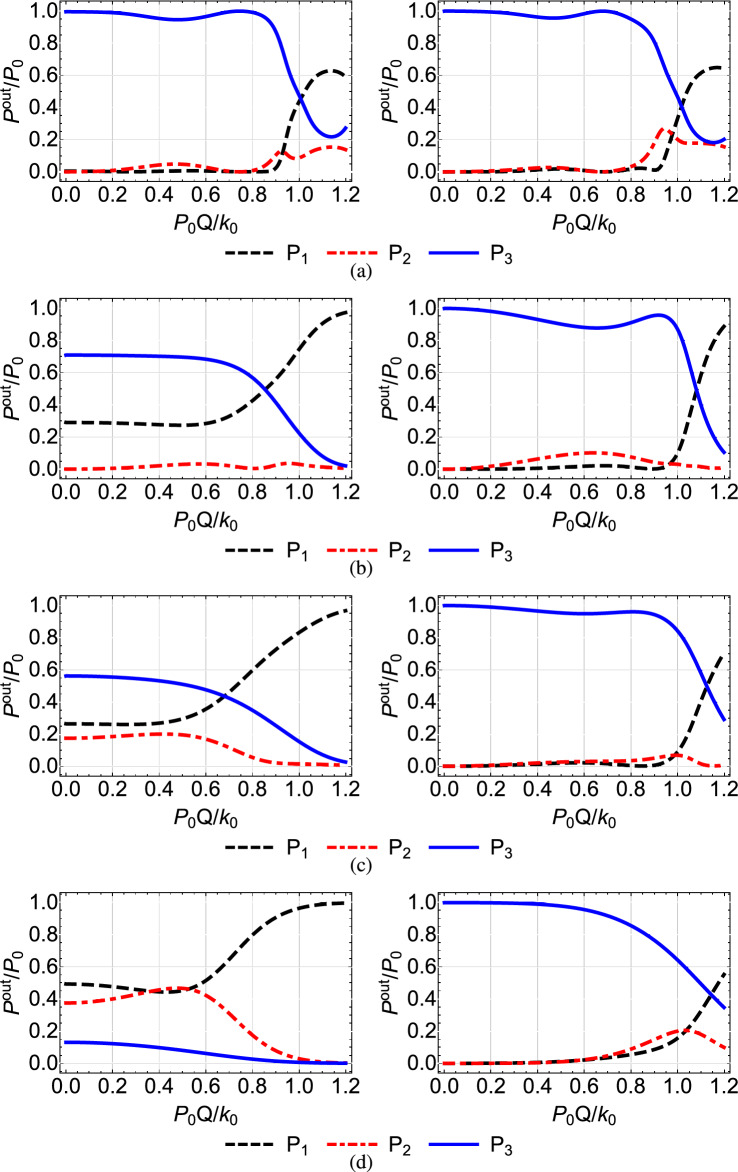
Normalized output power $P_i^{out}/P_0=P_i(Z)/P_0$ at each waveguide as a function of the normalized input power $P_0Q/k_0$ when $Q_1=Q, Q_2=0, Q_3=Q$, for the Gaussian (left column) and modified shortcut (right column) couplings displayed in the left and right columns of Fig. 2, respectively.

**Figure 8:**
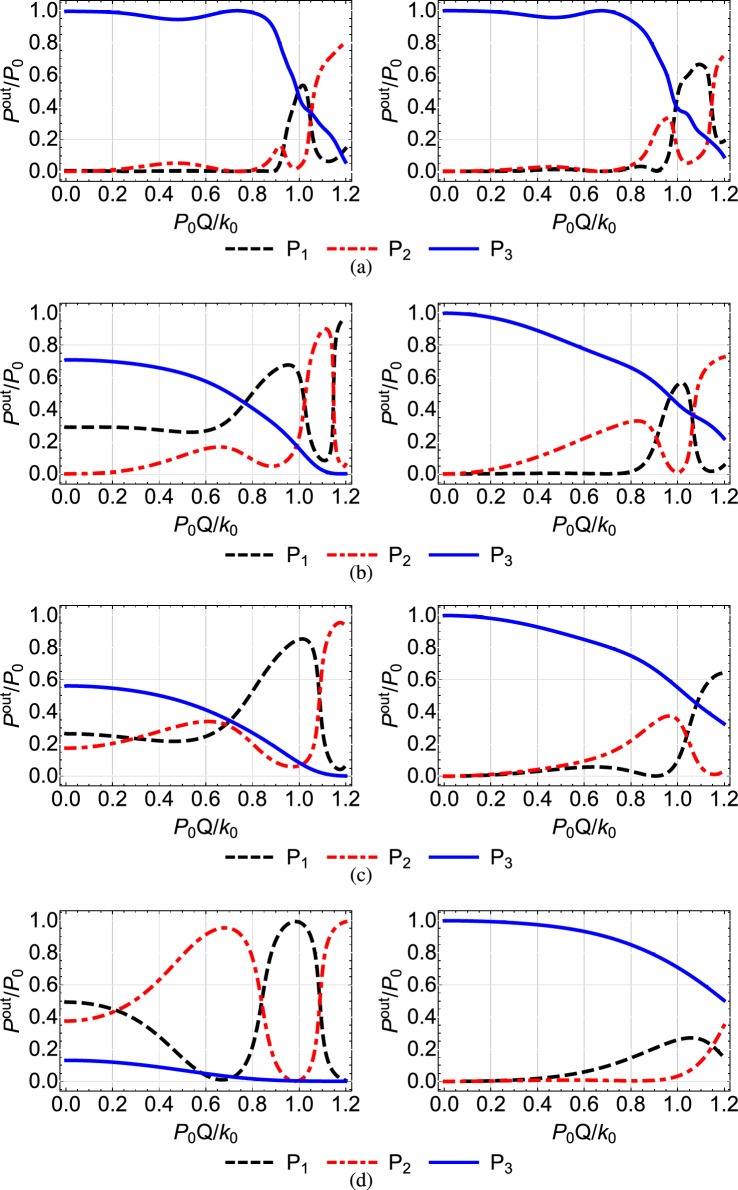
Normalized output power $P_i^{out}/P_0=P_i(Z)/P_0$ at each waveguide as a function of the normalized input power $P_0Q/k_0$ when $Q_1=Q, Q_2=Q, Q_3=Q$, for the Gaussian (left column) and modified shortcut (right column) couplings displayed in the left and right columns of Fig. 2, respectively.

In this section we test the performance of the previously derived modified couplings in the presence of nonlinearity in the waveguides, using in the transmission matrix (3) the modified coefficients ${\tilde{k}}_{12}, {\tilde{k}}_{23}$. The various configurations of the nonlinear coefficients $Q_i$ which we are going to study are shown in Table 1, along with the figure numbers where the corresponding results of numerical simulations are displayed. Note that in the considered configurations the coefficients $Q_{i}$ take only one of two values, *Q* if the corresponding waveguide is nonlinear, or zero if it is linear. In all the simulations we use propagation length $Z=30$ mm and maximum value of the Gaussian coupling coefficients $k_0=2$ mm$^{-1}$. For the nonlinear coefficients we use the parameters $A_i^{(eff)}=4$ $\mu$m$^2$, $n^{(2)}=1.5\times 10^{-13}$ cm$^2$/W, $\lambda =1.55$ $\mu$m, which lead to the value $Q=1.52\times 10^{-2}$ (mm $\cdot$ W)$^{-1}$^46^. Note that the value that we use for the nonlinear refractive index $n^{(2)}$ is typical for AlGaAs, see Refs.^41,42,45^. In Figs. 4, 5,6, 7 and 8 we plot for each configuration shown in Table 1 the normalized output power $P_i^{out}/P_0=P_i(Z)/P_0$ at each waveguide, as a function of the power $P_0$ inserted in the first waveguide, normalized by the quantity $k_0/Q=131.57$ W^46^. In each of these figures, in the right column we display the results corresponding to the shortcut couplings shown in the right column of Fig. 2, while in the left column we show for comparison the results obtained with the original Gaussian couplings of the left column in Fig. 2.

**Table 1:** Configuration of nonlinear coefficients in the triple waveguide and figure number where the corresponding results are displayed.

Configuration of nonlinear coefficients	Figures
$Q_1=Q_2=0$ and $Q_3=Q$	4
$Q_1=Q$ and $Q_2=Q_3=0$	5
$Q_1=Q_3=0$ and $Q_2=Q$	6
$Q_1=Q_3=Q$ and $Q_2=0$	7
$Q_1=Q_2=Q_3=Q$	8

A first observation which applies to all the figures is that, as we move from the top to the bottom row, thus the spatial extension of the coupling coefficients is reduced and the adiabaticity condition is not satisfied, the shortcut couplings outperform the original Gaussian couplings, since the power exiting the third waveguide (blue solid line) is higher. Only the results of the first rows are comparable, since in this case the adiabatic and shortcut couplings have slight differences, see the top row of Fig. 2. For the second to bottom rows, the superiority of shortcut couplings is eminent, indicating that these couplings can be exploited when the adiabaticity condition is violated, i.e. for smaller $k_{0}$ or shorter device length. A second overall observation is that for low input power $P_{0}Q/k_0<0.1$ the nonlinear effects do not significantly affect the performance of the device when the shortcut couplings are used and light is transferred to the third waveguide with high efficiency, confirming the robustness of the design method.

In Fig. 4, where $Q_1=Q_2=0$ and $Q_3=Q$, thus the nonlinearity is present only in the third waveguide, we observe for the modified couplings (right column) and higher values of the input power that the performance is reduced as we move from the first to the second row, since the power quickly entering the third waveguide affects more the transfer. The situation is somehow improved for shorter overlap between the coupling coefficients (third and fourth rows), since now the transfer in accomplished faster and the power in the third waveguide has a smaller effect. A similar behavior is observed in Fig. 5, where the nonlinearity is present only in the first waveguide, $Q_1=Q$ and $Q_2=Q_3=0$. Comparing Figs. 4 and 5 we notice that the efficiency achieved in the latter case is worse. The reason is that in this case the light inserted in the first waveguide immediately activates the nonlinearity in this waveguide, which affects the transfer from the beginning. In the case depicted in Fig. 4 the nonlinearity is activated later in the process, when light enters the third waveguide.

In Fig. 6, obtained for $Q_1=Q_3=0$ and $Q_2=Q$, we observe that when only the middle waveguide is nonlinear the transfer efficiency is not much affected by the input power, since during the propagation of light the middle waveguide is not excited enough for the nonlinear effects to take action, see the red dashed-dotted line in the right column of Fig. 3. Using the terminology of STIRAP, for this configuration the dark state remains an eigenstate of the system and the transfer of light takes place through it. However, as the coupling coefficients become narrower, the middle waveguide is excited more and more, see the right panels in Figs. 3(b,c,d), leading to a somehow degraded performance for higher input power, as depicted in the right column of Fig. 6.

In Fig. 7 where the first and third waveguides are nonlinear and the middle one is linear, $Q_1=Q_3=Q$ and $Q_2=0$, we obtain in general a better performance than the cases where only the first or only the third waveguide is nonlinear, depicted in Figs. 4 and 5, respectively. For example, in Fig. 7c we observe that under the shortcut couplings, we get a good transfer efficiency $P_{3}^{out}/{P_0} >0.9$ even for high values of the input power, with the nonlinearity significantly affecting the performance only for $P_0>0.9k_0/Q$. In Fig. 7d with the narrower couplings, we obtain very high efficiency levels for $P_0<0.4k_0/Q$. The improved performance compared to the cases where only $Q_1=Q$ or $Q_3=Q$ can be understood using the corresponding three-level picture of the system. The simultaneous presence of nonlinearity in both the first and third waveguides essentially reduces the effective two-photon detuning $\delta =Q_3|{\tilde{b}}_3(z)|^2-Q_1|{\tilde{b}}_1(z)|^2$.

Figure 8 corresponds to the case where the nonlinearity is present in all three waveguides, $Q_1=Q_2=Q_3=Q$. The performance of the shortcut couplings for large spatial extension is analogous to the previous case where the middle waveguide is linear ($Q_2=0$), compare the right panels in Figs. 7a and 8a. The reason is that in this case only a small amount of light passes through the middle waveguide, see the red dashed-dotted line in the right panel of Fig. 3a. Only for very large input power $P_0>k_0/Q$ this similarity breaks down. As the spatial extension of the shortcut couplings is decreased and the fraction of light through the middle waveguide is increased, the performance is further degraded compared to Fig. 7, see the right panel in Fig. 8b. But, as we further narrow the spatial extension of the couplings, we observe an improvement in the transfer efficiency and an increase in the threshold over which the nonlinear effects become important, see Figs. 8c and d. For example, in Fig. 8d we observe a very high efficiency up to $P_0=0.3k_0/Q$, while also for $P_0>0.9k_0/Q$ the performance is better compared to the case with larger spatial extent of the coupling coefficients, Fig. 8a. This behavior is similar to that observed in the right column of Fig. 4. It is due to the fact that narrower spatial extent of the couplings implies that the transition of light between the waveguides takes place in a smaller distance, so even for large values of the input power there is not enough excitation for the nonlinear coefficients to significantly affect the performance. For example, in the right panel of Fig. 3d, the spatial range over which the power in all the three waveguides has significant value, is very narrow.

**Figure 9:**
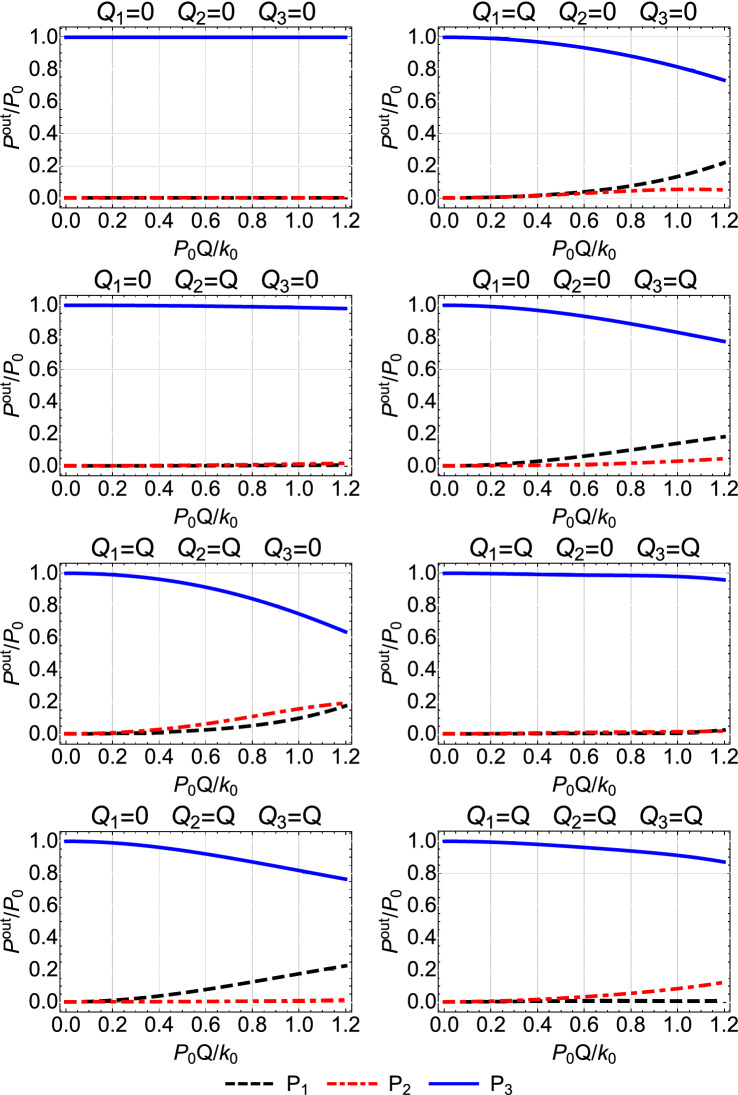
Normalized output power $P_i^{out}/P_0=P_i(Z)/P_0$ at each waveguide as a function of the normalized input power $P_0Q/k_0$ for various configurations and the narrow modified shortcut couplings displayed in the right panel of Fig. 10.

**Figure 10:**
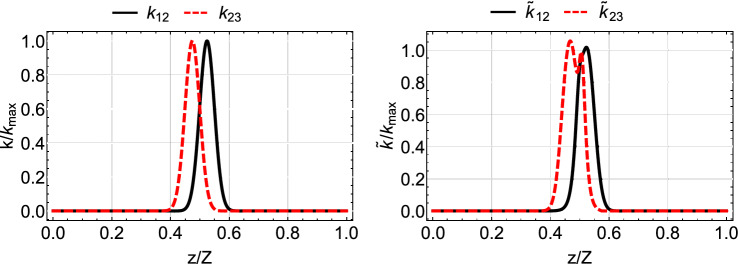
Gaussian (left column) and modified shortcut (right column) coupling coefficients for parameters $Z=30$ mm, $k_0=2$ mm$^{-1}$, $\tau =Z/40$ and $\zeta =Z/28$.

That last observation we made, that if the spatial extent of the coupling coefficients becomes narrow enough so the transfer of light between waveguides with the modified couplings takes place at a short enough distance, then the nonlinearity affects the performance less and less, is also demonstrated in Fig. 9. There we display the normalized power exiting the waveguides as a function of the normalized input power, for the very narrow shortcut couplings shown in Fig. 10 and for additional configurations of linear and nonlinear waveguides. As we previously noticed, the best efficiency is achieved for the symmetric configurations $Q_1=Q_3=Q, Q_2=0$, $Q_1=Q_2=Q_3=Q$, and $Q_1=Q_3=0, Q_2=Q$. The worst performance for higher input powers is obtained for the non-symmetric configurations $Q_1=Q, Q_2=Q_3=0$ and $Q_1=Q_2=Q, Q_3=0$, where the nonlinearity is present in the first (input) waveguide. A final interesting remark regarding the narrow modified couplings of Fig. 10 is that their maximum value slightly exceeds the maximum value $k_0$ of the Gaussian couplings; further decrease in the spatial extent of these coefficients results in even higher values, something that sets a lower limit on the device size reduction which can be achieved with shortcuts to adiabaticity.

## Conclusion

In conclusion, we used shortcuts to adiabaticity to design the variable couplings in a three-waveguide directional coupler which may contain nonlinear elements, in order to accomplish efficient light transfer between the outer waveguides for shorter device lengths, despite the presence of nonlinearity. We derived the shortcut couplings for the ideal case where all the waveguides are linear, for which a perfect transfer is guaranteed in theory, but we tested them for various combinations of linear and nonlinear waveguides in the device. We showed with numerical simulations that, in most configurations, high levels of transfer efficiency can be maintained even for large values of the input power, and for shorter lengths than those of conventional adiabatic devices. We also found that efficiency is improved for shortcut couplings with less spatial extent, since in this case the nonlinearity acts during a shorter range. The best performance was obtained for the symmetric configurations where the nonlinearity is present only in the middle or only in the two outer waveguides. The reason is that in the first case the dark state is still an eigenstate of the system, while in the second case the effective two-photon detuning is reduced. The worst performance for increasing input power was observed for the nonsymmetric configurations where the nonlinearity is present in the first (input) waveguide, thus it is immediately activated and undermines the transfer. The present work is expected to find application in research fields like optoelectronic computing and ultrafast light switching, where the fast and controlled light transmission inside a set of waveguides is a crucial task. Additionally, the reduction in the device size may be exploited for incorporating them in integrated optical systems, where a high density of waveguides is required.

## Data Availability

All data generated or analysed during this study are included in this published article.
